# Distal-Less Homeobox 5 Is a Therapeutic Target for Attenuating Hypertrophy and Apoptosis of Mesenchymal Progenitor Cells

**DOI:** 10.3390/ijms21144823

**Published:** 2020-07-08

**Authors:** John Twomey-Kozak, Salomi Desai, Wenguang Liu, Neill Y. Li, Nicholas Lemme, Qian Chen, Brett D. Owens, Chathuraka T. Jayasuriya

**Affiliations:** Department of Orthopaedics, Warren Alpert Medical School of Brown University and the Rhode Island Hospital, 1 Hoppin Street, Providence, RI 02903, USA; john_twomey-kozak@alumni.brown.edu (J.T.-K.); salomi_desai@brown.edu (S.D.); lwg890312@126.com (W.L.); li.neillyun@gmail.com (N.Y.L.); nicholas_lemme@brown.edu (N.L.); qian_chen@brown.edu (Q.C.); brett_owens@brown.edu (B.D.O.)

**Keywords:** DLX5, cellular hypertrophy, stem cells, apoptosis, osteoarthritis, gene therapy, progenitor cells, cartilage

## Abstract

Chondrocyte hypertrophy is a hallmark of osteoarthritis (OA) pathology. In the present study, we elucidated the mechanism underlying the relationship between the hypertrophy/apoptotic phenotype and OA pathogenesis in bone marrow-derived mesenchymal stem cells (BM-MSCs) via gene targeting of distal-less homeobox 5 (DLX5). Our primary objectives were (1) to determine whether DLX5 is a predictive biomarker of cellular hypertrophy in human osteoarthritic tissues; (2) To determine whether modulating DLX5 activity can regulate cell hypertrophy in mesenchymal stem/progenitor cells from marrow and cartilage. Whole transcriptome sequencing was performed to identify differences in the RNA expression profile between human-cartilage-derived mesenchymal progenitors (C-PCs) and bone-marrow-derived mesenchymal progenitors (BM-MSCs). Ingenuity Pathway Analysis (IPA) software was used to compare molecular pathways known to regulate hypertrophic terminal cell differentiation. RT-qPCR was used to measure DLX5 and hypertrophy marker COL10 in healthy human chondrocytes and OA chondrocytes. DLX5 was knocked down or overexpressed in BM-MSCs and C-PCs and RT-qPCR were used to measure the expression of hypertrophy/terminal differentiation markers following DLX5 modulation. Apoptotic cell activity was characterized by immunostaining for cleaved caspase 3/7. We demonstrate that DLX5 and downstream hypertrophy markers were significantly upregulated in BM-MSCs, relative to C-PCs. DLX5 and COL10 were also significantly upregulated in cells from OA knee joint tissues, relative to normal non-arthritic joint tissues. Knocking down DLX5 in BM-MSCs inhibited cell hypertrophy and apoptotic activity without attenuating their chondrogenic potential. Overexpression of DLX5 in C-PCs stimulated hypertrophy markers and increased apoptotic cell activity. Modulating DLX5 activity regulates cell hypertrophy and apoptosis in BM-MSCs and C-PCs. These findings suggest that DLX5 is a biomarker of OA changes in human knee joint tissues and confirms the DLX5 mechanism contributes to hypertrophy and apoptosis in BM-MSCs.

## 1. Introduction

Osteoarthritis (OA) is a leading cause of disability and functional impairment in the United States [1,2]. As the articular cartilage is regularly exposed to biomechanical forces from joint impact and torsion, focal defects via traumatic injury and chronic degeneration are common [3]. Because cartilage is avascular, its capacity for healing following injury is minimal [4]. Consequently, most cartilage injuries will linger with a significantly increased risk of the early onset of OA [5,6]. Further, studies show that cell hypertrophy is a hallmark of osteoarthritis (OA). It is also known that the occurrence of cell hypertrophy is a challenge that needs to be overcome in order to improve the current cell-mediated cartilage defect repair strategies, such as Autologous Chondrocyte Implantation (ACI) and microfracture surgery.

In lieu of this challenge, interest has shifted towards the development of biologic cell therapies designed to stimulate cartilage and fibrocartilage healing [7]. Ideal characteristics of cells to be used for cartilaginous tissue engineering/repair are: (1) sufficient bioavailability, (2) high proliferative capacity (to quickly fill defects) [8], (3) robust chondrogenic capability (to resynthesize lost/damaged proteoglycan and collagen networks) [9], and (4) resistance to cellular hypertrophy (to maintain healthy tissue homeostasis and avoid OA) [10]. Bone-marrow-derived mesenchymal stem cells (BM-MSCs) have been extensively researched in preclinical models of cartilage tissue repair as these cells predominantly meet the first three criteria [11]. However, BM-MSCs have a tendency to become hypertrophic [12], especially when driven down the chondrogenic lineage [13]. During late-stage chondrogenesis, BM-MSCs exhibit increased gene expression of common cartilage hypertrophy-ossification markers such as type X collagen (*COL10*), alkaline phosphatase (*ALP*), bone gamma-carboxy-glutamic acid-containing protein (*BGLAP*), and runt-related transcription factor-2 (*RUNX2*) [12,13]. This poses a significant challenge, since hypertrophy and senescence are believed to precede the ossification and mineralization that occurs along the endochondral ossification pathway [12,14]. 

It has been reported that mesenchymal progenitors that come from articular cartilage, referred herein as cartilage-derived progenitor cells (C-PCs), exhibit similar phenotypic qualities of BM-MSCs, with the exception that C-PCs are resistant to hypertrophic changes [15,16]. In the present study, we performed comparative large data transcriptome analysis between C-PCs and BM-MSCs in order to identify differently expressed target genes that may explain why BM-MSCs are more susceptible to cellular hypertrophy than C-PCs. Our goal was to determine whether we can use this information to genetically modulate BM-MSCs to resist terminal differentiation and hypertrophy while still preserving their capacity for chondrogenic differentiation. Overall, we discovered that distal-less homeobox 5 (DLX5), which is a bone-morphogenic protein 2 (BMP-2)-inducible transcription factor that is upstream of late-stage chondrogenesis and hypertrophy markers, is significantly upregulated in BM-MSCs. We report here that knocking down *DLX5* in BM-MSCs reduces the expression of common hypertrophy markers while allowing these cells to still retain their chondrogenic capacity. Conversely, we report that overexpression of *DLX5* in C-PCs compromises the ability of these cells to resist hypertrophy and stimulates increased apoptotic pathway activation. This study provides first-time evidence that DLX5 is a critical regulator of hypertrophy and apoptosis in mesenchymal stem/progenitor cells. This finding is significant because it suggests that modulating DLX5 may provide a novel avenue to prevent hypertrophy and terminal differentiation in cells used in cartilage tissue repair applications. 

## 2. Results

### 2.1. C-PCs Are Mesenchymal Progenitor Cells that Resist Cell Hypertrophy

**Figure 1:**
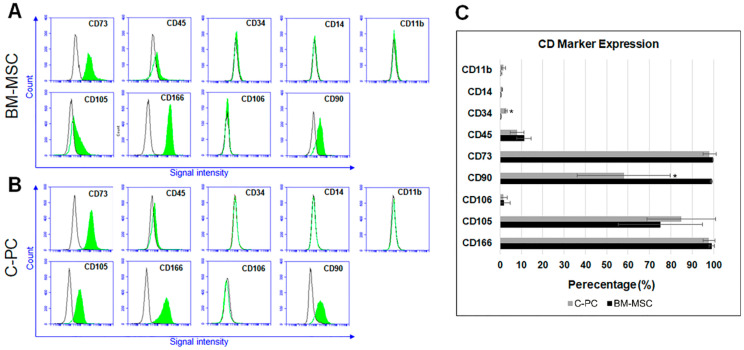
Immunotypic comparison of cartilage-derived progenitor cells (C-PCs) and bone marrow-derived mesenchymal stem cells (BM-MSCs). (**A**) The cell surface marker profile of BM-MSCs and (**B**) C-PCs as determined using flow cytometry. Color filled peaks indicate the percentage of cells that stained positively for each antibody. Empty, noncolored peaks represent the results of cells that were stained with isotype control antibodies. (**C**) Percentage of cells that stained positively for each tested antibody is shown in a representative bar graph. *, *p* ≤ 0.05, compared to respective BM-MSC groups and (*N* = 3 replicate experiments).

It has been previously surmised that the C-PC is an ideal cell type for cartilaginous tissue repair/regeneration based on: (1) their natural placement within articular cartilage tissue [17], (2) their functional phenotype (i.e., chondrogenic capacity, resistance to terminal differentiation) [16,18]. It is demonstrated that C-PCs exhibit low basal expression of cell hypertrophy markers during in vitro cell culture [16] and ex vivo tissue coculture, in comparison to BM-MSCs [18]. Despite differences in their functional phenotype, C-PCs and BM-MSCs share similar immunophenotypic cell surface marker profiles (Figure 1). Veritably, C-PCs satisfy the criteria outlined by the International Society for Cellular Therapy for characterizing mesenchymal stromal/stem cells [18,19]. Like BM-MSCs, C-PCs do not exhibit common hematopoietic stem cell surface markers CD34 and CD45; nor do they exhibit mononuclear peripheral blood-derived cell surface markers CD14 and CD11b (Figure 1A–C). They also lack endothelial cell surface marker CD106/VCAM (Figure 1B,C). C-PCs positively express immunophenotypic cell surface markers consistent with that of all mesenchymal stem cells including CD90, CD105, CD73, and CD166 (Figure 1B,C). The only notable difference among the tested cell surface markers in C-PCs and BM-MSCs was that the expression level of CD90 was noticeably reduced in C-PCs compared to BM-MSCs; however, both cell types positively expressed this marker (although at quantifiably different levels—57% in C-PCs, 98% in BM-MSCs, Figure 1C).

### 2.2. Transcriptome Analysis Identifies DLX5 as a Regulator of Terminal Chondrocyte Differentiation and Hypertrophy that is Elevated in Human BM-MSCs

**Figure 2:**
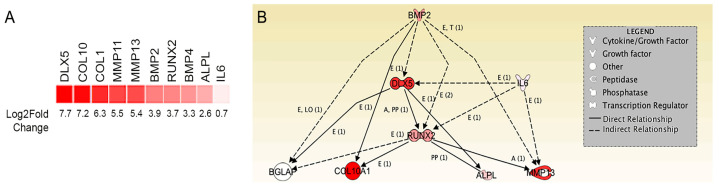
Network of hypertrophy, ossification, and proinflammatory genes that are differently expressed between BM-MSCs and C-PCs. (**A**) Heat map of differential gene expression in BM-MSCs. Results are displayed in increasing order of expression levels based on log2 fold change. (**B**) Ingenuity Pathway Analysis (IPA) software analysis of BM-MSC RNA Seq data illustrates the central role of DLX5 in hypertrophy and ossification pathway networks. The figure legend displays the biomolecular identity of each molecule. Within direct pathways, E = regulates expression levels, T = affects transcription levels, PP = protein to protein interactions, LO = left out but indicates gene affects expression.

RNA sequencing and molecular pathway analysis were performed using Ingenuity Pathway Analysis (IPA) software to compare the distinct transcriptomes of C-PCs and BM-MSCs. Our goal here was to identify differently expressed upstream regulators of know markers of terminal chondrocyte differentiation and hypertrophy that might explain the phenotypic dissonance between C-PCs and BM-MSCs. In order to do this comparison, we used total RNA from primary human BM-MSCs and total RNA from an established C-PC cell line (CPCL3) that we had generated and extensively characterized in a previous study [18]. Using IPA, we stratified our parameters to output hypertrophy, ossification, and cartilage catabolism-related gene networks that were differently expressed between these two cell types. Our analysis indicated that BM-MSCs expressed significantly higher levels of multiple key markers of terminal chondrocyte differentiation (*COL10, RUNX2*), mineralization/bone development (*ALPL, BMP-2, BMP-4*) and cartilage remodeling (*MMP-11, MMP-13*, Figure 2A, Table S1). Within these selected parameters, the marker that was most elevated was however *DLX5*, which is an upstream regulator of the majority of the aforementioned targets (Figure 2B). DLX5 is a BMP-responsive transcription factor that is reported to regulate chondrocyte hypertrophy [20,21,22,23]. Pathway analysis indicated that DLX5 is directly upstream of hypertrophy and ossification-associated genes *BGLAP, ALPL, RUNX*2, and *COL10* and that it is also an indirect regulator of *MMP-13* (Figure 2B).

### 2.3. DLX5 Is Elevated in OA Cartilage Cells and Correlates Positively with COL10 Expression

**Figure 3:**
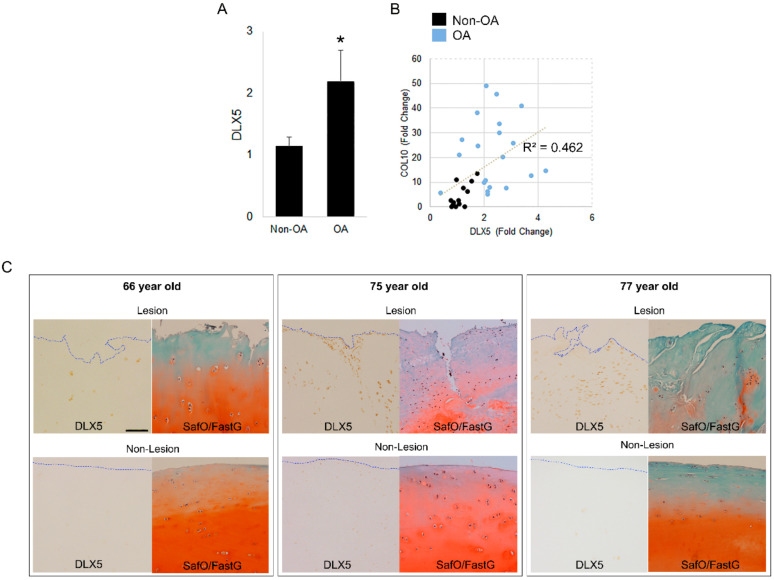
Distal-less homeobox 5 (*DLX5*) is elevated in osteoarthritis (OA) chondrocytes and OA cartilage lesions. (**A**) RT-qPCR results of *DLX5* and *COL10* mRNA expression fold change in nonarthritic human chondrocytes and osteoarthritic (OA) chondrocytes. OA chondrocytes have significantly higher *DLX5* expression than nonarthritic chondrocytes. N ≥ 3 patient samples in each group. *, *p ≤* 0.05. (**B**) *DLX5* and *COL10* mRNA expression levels in patient chondrocytes show a positive correlation (R^2^ = 0.462). Each single data point represents an independent biological replicate from each patient. Non-OA chondrocyte cultures from three patients and OA chondrocyte cultures from four patients were used to perform analysis. There were N ≥ 4 biological replicates analyzed per patient. (**C**) DLX5 immunostaining in human OA cartilage lesions and intact regions (non-lesions) in three different patients. DLX5 protein is stained brown. DLX5 protein expression is visibly prominent in cells from cartilage lesions. Comparatively less intense staining was evident along intact non-lesion regions of OA cartilage. The blue dotted line indicates the periphery (edge) of the articular surface. Safranin-O/Fast green staining is performed on sister sections to help confirm OA pathology in these cartilage tissues. Scale bar = 100 µm.

Chondrocyte hypertrophy is a hallmark of osteoarthritic changes in joint cartilage. We compared the expression of *DLX5* in human-cartilage-derived cells from four different OA patients (age 72F, 72F, 66F, and 66M) and those isolated from three different patients that did not present with OA (ages 15F, 18F, 42F). Cells from OA cartilage exhibited higher expression of DLX5 as well as downstream terminal hypertrophic differentiation marker *COL10* (Figure 3A,B). Interestingly, the relative mRNA expression level of *DLX5* shows a positive correlation with COL10 (R^2^ = 0.462), supporting our hypothesis that *DLX5* drives *COL10* expression (Figure 3B). These findings suggest that DLX5 is an OA-associated biomarker that is linked to hypertrophic cellular changes that are a hallmark of this degenerative joint disease.

We also performed DLX5 immunostaining in OA patient-derived knee articular cartilage that was taken from arthritic tissue lesions, as well as intact areas that did not have signs of degeneration (non-lesions, Figure 3C). Cartilage from three different OA patients (ages 66F, 75M, and 77M) was used for staining. While some DLX5 staining was visible in non-lesioned areas of OA cartilage, the strongest staining was visible in the arthritic lesions. Taken together, these findings also suggest that DLX5 expression is an indicator of cellular hypertrophy and cartilage degeneration during OA.

### 2.4. DLX5 Knockdown Attenuates Hypertrophy and Decreases Apoptotic Cell Fate without Deterring Chondrogenesis in BM-MSCs 

**Figure 4:**
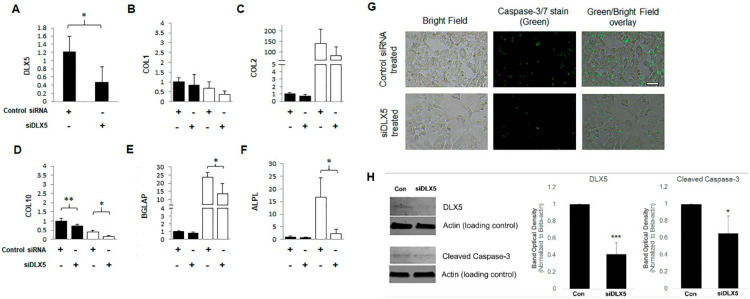
DLX5 knockdown results in reduced expression of hypertrophy/ossification-related genes and decreased apoptosis in BM-MSCs. Relative mRNA expression levels of (**A**) *DLX5*, early chondrogenesis markers (**B**) *COL1* and (**C**) *COL2*, terminal hypertrophic differentiation and ossification-related markers (**D**) *COL10*, (**E**) *BGLAP* and (**F**) *ALPL* in DLX5 siRNA knockdown BM-MSCs vs Control-scrambled siRNA BM-MSCs. The mRNA expression results are displayed at the basal level (“Noninduced” black-colored bars in the graph) and then again after undergoing chondrogenesis (“Chondro-induced” white-colored bars in the graph) to confirm the retention of the genotype. *N* ≥ 3; *, *p* ≤ 0.05; **, *p* ≤ 0.01 relative to control BM-MSCs. (**G**) Caspase-3/7 immunofluorescent staining of DLX5 siRNA knockdown BM-MSCs vs. Control-scrambled siRNA BM-MSCs. Scale = 250 µm. (**H**) Western blot analysis of DLX5, cleaved caspase-3, and the respective actin loading controls in DLX5 siRNA knockdown BM-MSCs and Control-scrambled siRNA BM-MSCs. Bar graphs represent the mean densitometric intensities of bands, normalized to Actin loading control bands. Measurements were made using ImageJ software. *N* = 3; *, *p* ≤ 0.05; ***, *p* ≤ 0.005.

In order to confirm the mechanism by which BM-MSCs become hypertrophic, we transiently knocked down the DLX5 gene in BM-MSCs using RNA interference, and examined the subsequent effect of canonical markers of cell hypertrophy. Quantitative RT-PCR analysis confirmed that *DLX5* mRNA expression was knocked down to approximately 40% efficiency in BM-MSCs transfected with small interfering RNA specifically targeting *DLX5* (siDLX5, Figure 4A). Neither fibrochondrocyte marker *COL1* nor chondrocyte marker *COL2* mRNA levels were significantly altered by *DLX5* knockdown (Figure 4B,C). Such was the case when cells were cultured in growth medium (referred to as “Noninduced” in Figure 4A–F to represent basal culture conditions) as well as when cells were cultured in chondrogenesis inducing medium (referred to as “Chondro-induced” in Figure 4A–F to represent chondrogenic culture conditions) for 72 h. However, upon chondrogenesis induction, the resulting expression of hypertrophy and terminal chondrocyte differentiation markers *COL10, ALPL,* and *BGLAP* were all significantly decreased in the *DLX5* knockdown siRNA treated group compared to the scrambled siRNA treated control group (Figure 4D–F). 

As a second method to verify that inhibiting DLX5 in BM-MSCs effectively inhibits hypertrophic changes in these cells, we investigated whether *DLX5* knockdown affects caspase activity, which is associated with cellular apoptosis. We did this because apoptosis is a known hallmark of terminal hypertrophic differentiation of chondrocyte lineage cells during their development. We observed that transient *DLX5* knockdown in BM-MSCs achieved a modest but noticeable reduction in apoptotic cells as indicated by the decreased fluorescent intensity of activated caspase (Figure 4G). Western blotting confirmed that cleaved caspase-3 was significantly reduced in *DLX5* knockdown BM-MSCs, relative to scrambled siRNA treated control BM-MSCs. Taken together, these results indicate that suppressing *DLX5* is sufficient to dissuade the terminal chondrocyte differentiation program and apoptosis; however, the reduction of *DLX5* did not seem to affect the expression of key markers of early-stage chondrogenesis.

### 2.5. Elevated DLX5 Expression Stimulates Cell Hypertrophy and Apoptosis in C-PCs

**Figure 5:**
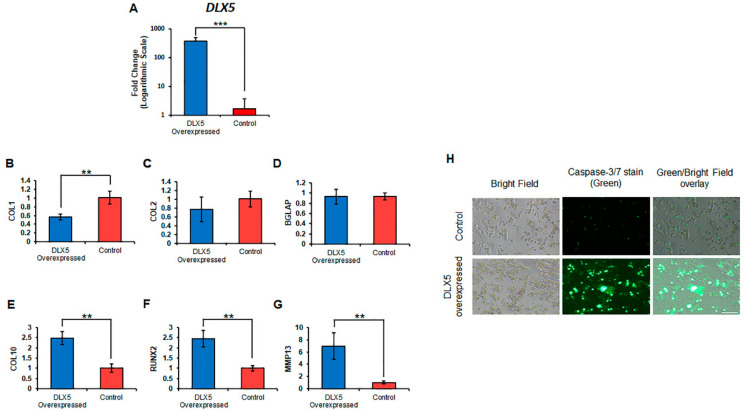
Overexpression of DLX5 in C-PCs stimulate hypertrophy, ossification, catabolism markers and increases apoptosis. (**A**) Relative mRNA expression levels of DLX5 gene, chondrogenic markers (**B**) COL1, (**C**) COL2 as well as hypertrophy and ossification-related markers (**D**) BGLAP, (**E**) COL10, (**F**) RUNX2, and catabolic proteinase (**G**) MMP13 in DLX5 overexpressed C-PCs vs. control C-PCs. *N* ≥ 3; **, *p* ≤ 0.01; ***, *p* ≤ 0.005 relative to control C-PCs. (**H**) Caspase-3/7 immunofluorescent staining of DLX5 overexpressed C-PCs vs. control C-PCs. Scale = 250 µm.

C-PCs exhibit significantly lower basal expression of *DLX5* transcripts, compared to BM-MSCs (see again Figure 2A). We hypothesized that this cellular characteristic might help to explain why C-PCs are resistant to terminal hypertrophic differentiation. In order to further test this hypothesis, we next investigated whether increasing *DLX5* expression in C-PCs would be sufficient to stimulate hypertrophic changes and apoptosis in these cells. The human DLX5 transgene was overexpressed in the stable C-PC cell line CPCL3 using an adenoviral vector (Ad-h-DLX5). Infection with Ad-h-DLX5 increased *DLX5* transcripts ~231-fold in CPCL3 (Figure 5A). *DLX5* overexpression in CPCL3 resulted in a statistically significant reduction in *COL1* expression (Figure 5B); however, *COL2* levels were unaffected (Figure 5C). As expected, *DLX5* overexpression elevated terminal chondrocyte differentiation and hypertrophy marker expression. This included a 2.5-fold increase in *COL10* (Figure 5E), and a 2.4-fold increase in *RUNX2* (Figure 5F). Surprisingly, *BGLAP* expression did not appear to be affected significantly by *DLX5* overexpression in this experiment. This can be explained since mRNA expression levels of *BGLAP* are already minimal in C-PCs (i.e., raw CT-value ~34, as detected by RT-qPCR). Further, mRNA levels of the collagen-degrading catabolic enzyme MMP13 exhibited a seven-fold expression increase in the *DLX5* overexpression group (Figure 5G). Our results also demonstrated that elevating DLX5 levels in C-PCs directly results in a significant increase of apoptotic cells characterized by the increased fluorescent intensity of activated caspase-3/7 (Figure 5H). Overall, these findings suggest that DLX5 is a positive regulator of terminal hypertrophic differentiation and apoptosis in mesenchymal progenitor/stem cells. 

## 3. Discussion

In this investigation, DLX5 was identified as a positive regulator of cell hypertrophy and apoptosis. DLX5 inhibition in BM-MSCs was found to significantly reduce cellular hypertrophy without inhibiting their chondrogenic potential. In contrast, overexpression of DLX5 in C-PCs demonstrated a significant increase in cellular hypertrophy markers. These genetic knockdown and overexpression experiments were performed to confirm that the DLX5 mechanism contributes to cell hypertrophy and apoptosis. Clinically, cartilage samples taken from patients of varying ages and pathologies demonstrated a rise in DLX5 given greater osteoarthritic pathologies. Taken together these results demonstrate a cellular hypertrophy regulator, DLX5, whose inhibition may be crucial in improving cell-mediated cartilage tissue repair. 

Type X collagen is considered to be the most common marker for BM-MSC and chondrocyte hypertrophy, as deposits serve as the framework for subsequent ossification in the form of matrix calcification [24,25]. Further, cell hypertrophy results in increased cell size/volume and elevated expression of *MMP13, ALPL,* vascular endothelial growth factor (*VEGF*), and *BGLAP*, which are downstream targets of RUNX2 [12,14,26,27,28]. This was also a phenomenon we observed in our study, as patient OA chondrocytes that were in the vicinity of arthritic lesions appeared to have significantly larger cell volume and greater DLX5 production. Although it is not well understood if the expression of such terminal hypertrophy markers directly results in increased cell volume or vice versa, it is evident that a change in physical volume will directly affect cell function [29,30,31]. When this occurs, the cellular matrix is less able to counter osmotic swelling forces leading to weakening and breakdown of the matrix itself [14,32]. It is also crucial to note that the hypertrophy related changes listed above are not limited to terminal chondrocyte differentiation, but occur during pathological osteoarthritis [33,34,35,36]. Thus, using a mesenchymal cell type that is prone to hypertrophy and terminal differentiation for cartilaginous tissue regeneration is less than ideal, as it can potentially accelerate osteoarthritis. 

Here, we demonstrate that BM-MSCs express significantly elevated levels of numerous catabolic and hypertrophy-associated genes with *DLX5* being the center of this hypertrophic pathway. Expression of hypertrophy and ossification-associated markers *COL10, ALPL* and *BGLAP* were all significantly reduced in the BM-MSC DLX5 knockout group, in both basal state (in normal growth medium) and chondrogenic conditions (in chondrogenic-supplemented medium). It should be noted however that the knockdown of *DLX5* to 40% efficiency was insufficient to cause a reduction in *RUNX2* mRNA expression in BM-MSCs [37]. However, later we showed that overexpression of *DLX5* by 231-fold in C-PCs, did result in *RUNX2* expression, confirming that DLX5 regulates RUNX2. Further, mRNA expression level differences of chondrogenic genes *COL2A1/COL1A1* were statistically insignificant between *DLX5* knockdown and control groups regardless of the medium used, thereby demonstrating that DLX5 attenuation does not impact early-stage chondrogenesis. *DLX5* knockdown decreased apoptotic activity in BM-MSCs, as indicated by decreased active/cleaved caspase staining and protein quantification. These findings have important ramifications for cell-based cartilaginous tissue repair, as progression to functional neo-cartilaginous tissue formation would require significant chondrogenic capacity and resistance to cell hypertrophy as well as apoptosis.

In C-PCs, which inherently express low levels of *DLX5*, overexpression of *DLX5* led to greater hypertrophic markers and apoptotic activity in C-PCs. While chondrogenesis marker *COL2* remained relatively consistent, the expression of hypertrophic markers *COL10, RUNX2,* and *MMP-13* was significantly elevated. Active caspase-3/7 immunofluorescence staining in overexpressing *DLX5* directly increased levels of apoptosis. Nonapoptotic roles for caspase-3 and caspase-7 have been described and identified during the process of ossification, bone development, and remodeling in mice. Miura et al. reported caspase-3-deficient mice exhibited gross skeletal defects and bone malformation [38]. BM-MSCs from these caspase-3-deficient mice, revealed an impaired osteogenic capacity. Svandova et al. reported similar nonapoptotic functions for caspase-7 whereby activated caspase-7 was detected in intramembranous bone that (formed by the ossification of the mesenchyme) and endochondral bones (formed by cartilage pathways) [39]. Further, Janečková et al. demonstrated an increasing gradient of caspase-positive cells from the resting to ossification zone in embryonic mouse forelimbs and decreased mRNA expression of 46 osteogenic genes after 7 days of caspase inhibition [40]. These studies suggest that caspases have nonapoptotic functions in osteogenesis. Specifically, activated caspase-3 and caspase-7 play a direct role in the ossification addition to their established apoptotic function. Therefore, as caspase-3/7 staining was decreased when the DLX5 gene was knocked down and increased with *DLX5* overexpression, it is evident that in addition to its central role in the hypertrophy program, DLX5 may also play a key role in the apoptosis and ossification pathways.

In the current literature, it is surmised that the degeneration of cartilage that occurs in OA may be a result of fewer numbers of chondrocytes [41]. These cells fail to regenerate and remodel the cartilage—thus sacrificing any chondroprotective or chondroregenerative role—and instead undergo terminal differentiation and apoptosis. It has been postulated that this process of apoptosis is linked to OA pathogenesis. Namely, Hwang and Kim reported that in late-stage OA, cartilage exhibits the quintessential features of chondrocyte hypertrophy and imminent death: hypocellularity and lacunar emptying [42]. This is significant because it provides evidence that chondrocyte death is a central feature of OA progression and therefore that apoptosis may be linked to OA changes in the knee cartilage. Findings presented in our study demonstrate that *DLX5* is expressed at a higher level in OA chondrocytes than nonarthritic chondrocytes, and that its expression has a positive correlation with *COL10* expression (a canonical marker of cell hypertrophy that is downstream of DLX5). Furthermore, we observed that DLX5 staining is significantly elevated in cells surrounding OA lesions. Taken together, these results indicate that DLX5 may contribute to cellular hypertrophy and catabolism that is a hallmark of OA.

**Figure 6:**
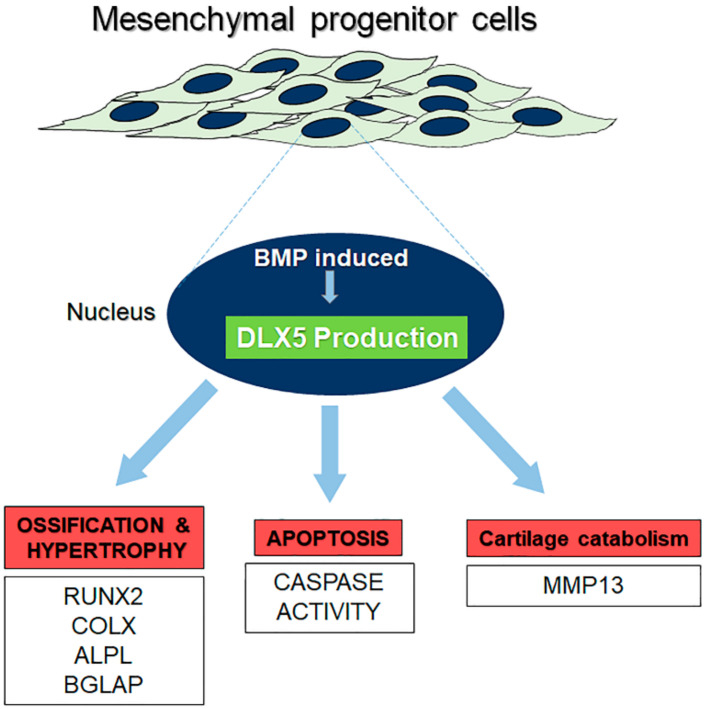
Proposed schematic of DLX5 function in mesenchymal progenitor cells.

We also quantified in this study the expression of DLX5 in cells isolated from OA patients in order to investigate its correlation with cell hypertrophy in OA. Our results showed that OA patients expressed elevated levels of both *DLX5* and *COL10* in their chondrocytes, compared to nonarthritic chondrocytes. Our findings demonstrate that *DLX5* is not only involved in the terminal hypertrophic differentiation pathway, but that *DLX5* mRNA expression levels also positively correlate with the osteoarthritic phenotype. Based on the findings of the present study, a comprehensive summary schematic describing the proposed role of DLX5 in regulating mesenchymal progenitor cell fate is illustrated in Figure 6. 

Our future studies will focus on investigating the efficacy of DLX5 deficient BM-MSCs in cartilage and fibrocartilage tissue repair. We anticipate that using adenovirus (AD) or adeno-associated virus (AAV) mediated knockdown of *DLX5* is the most practical approach for developing this novel strategy for clinical translation, as both methodologies are currently clinically relevant modalities of gene delivery/modification [43,44]. 

## 4. Methods

### 4.1. Human Cell and Cell Line Culture

Human tissue samples were procured with Rhode Island Hospital (RIH) Institutional Review Board approval (Code: 007017, approved 5 May 2017) Healthy human cartilage was obtained from knee arthroscopy surgeries of three patients (ages 15F, 18F, 42F) in which injured, but not osteoarthritic, articular cartilage was collected using a GraftNet™ Autologous Tissue Collectors (Arthrex, Naples, FL, USA). OA articular cartilage was collected from patients undergoing total knee replacement surgery. A total of four different patients (ages 72F, 72F, 66F, and 66M) were used for mRNA quantification. Cartilage samples collected from three more OA patients (ages 66F, 75M, and 77M) were used for histological analysis. Samples were washed thoroughly with 1× Hanks’ balanced saline solution (HBSS) and processed. Tissue was diced and digested in Pronase (Roche, Indianapolis, IN, USA; 2.0 mg/mL in 1× HBSS) for 30 min at 37 °C in a shaker. Tissue was further digested in Type IIA Crude Bacterial Collagenase (Sigma-Aldrich, St. Louis, MO, USA; 1.0 mg/mL) for 12 h at 37 °C in a shaker. Cells were passed through a nylon strainer (45 µm pore size) and washed 3× in Dulbecco’s modified Eagle’s medium containing 10% fetal bovine serum (DMEM+). 

We used a stable human C-PC cell line (CPCL3) that was previously generated and thoroughly characterized in our laboratory [18] in this study. Human BM-MSCs were purchased from ATCC (Manassas, VA, USA). We utilized BM-MSCs that were also stabilized using the pRetro-E2 SV40 construct (Applied Biomaterials Inc., Richmond, BC, Canada), according to the manufacturer’s protocol, in order to complete all DLX5 modulation experiments. PRetro-E2 SV40 is the same construct that was used to stabilize human C-PCs [18]. C-PCs were maintained in DMEM with 10% FBS, 1% Pen Strep, 100 mM HEPES, 2 mM L-glutamine, 0.1 mM ascorbic acid, 0.1 mM sodium pyruvate, and 2.7 µM L-glucose (DMEM++) in a humidified incubator at 37 °C and 5% COBM-MSCs were maintained in commercially available StemPro MSC medium (ThermoFisher, Carlsbad, CA, USA). 

### 4.2. mRNA Expression Analysis via RT-qPCR

Total RNA was isolated from cells via RNAqueous Kit (Ambion, Austin, TX, USA) and MagMAX-96 Total RNA Isolation kit (Thermo Fisher Scientific, Waltham, MA, USA) according to the manufacturer. RNA was reverse transcribed to cDNA using iScript cDNA Synthesis Kit (Bio-Rad, Hercules, CA, USA) according to the manufacturer. Gene expression analysis was then performed using standard quantitative real-time polymerase chain reaction (RT-qPCR). Table 1 lists primers used for conducting the analysis. Expression levels were calculated using the delta-delta Ct (ΔΔCt) method. *X* = 2^−ΔΔCt^, in which ΔΔCt = (Ct_Exp target gene_ − Ct_Exp house-keeping gene_) − (Ct_Ctl target gene_ − Ct_Ctl house-keeping gene_) and *X* = relative transcript; Ct_Exp_ = Ct of experimental group, Ct_Ctl_ = Ct of control group. Beta-Actin (β-Actin) was used as a housekeeping gene for normalization.

### 4.3. Immunophenotyping of BM-MSC and C-PC Cultures

Characterization of BM-MSCs and C-PCs was performed using flow cytometry analysis. Antibodies used for flow and their descriptions are listed in Table 2 IgG isotype control antibodies were purchased from Miltenyi Biotech Inc., San Diego, CA, USA. Cells (1.0 × 10^6^) were detached from culture dishes, washed in 5 mL of 1× PBS, centrifuged at 300 g and resuspended in 100 μL of buffer (1× PBS, 0.5% bovine serum albumin, and 2 mM EDTA). The fluorochrome-conjugated monoclonal antibody was added (10 μL), mixed gently, and incubated in the dark at 4 °C for 10 min. Excess antibody was washed using 1.0 mL of 1× PBS. Stained cells were resuspended in 500 μL of 1× PBS and analyzed using Accuri C6 Flow Cytometer (BD Biosciences, San Jose, CA, USA).

### 4.4. RNA Sequencing and Transcriptome Analysis

Total RNA was extracted and purified from C-PCs and BM-MSCs using Qiagen miRNeasy kit. Pure total RNA samples were sent to GENEWIZ (South Plainfield, NJ, USA), and they performed library construction, template preparation, and RNA sequencing. Library preparation was performed using Poly(A)+ RNA selection. Biological triplicate samples (*N* = 3) per group were sent out for RNA sequencing. Downstream differential expression analysis was performed by GENEWIZ using DESeq2 software. We used Ingenuity Pathway Analysis (IPA) software to analyze RNA seq data sets. Inclusion criteria for the generated canonical cell hypertrophy pathway networks were based on an IPA-generated confidence score. Further, the genes included in these networks met two pathway inclusion criteria that included: high log2fold change expression values and statistically significant *p*-values (*p* < 0.001) to determine the probability of association. 

### 4.5. DLX5 Knockdown and Overexpression Experiments

DLX5 knockdown (KD) in BM-MSCs was achieved by transfecting these cells with ON TARGETplus DLX5 SMARTpool siRNA (Dharmacon, Lafayette, CO, USA). Nontargeting siRNA control (Dharmacon, Lafayette, CO, USA) was used to transfect BM-MSCs in the respective control group. First, 5 × 10^4^ BM-MSCs were seeded per well in a 12-well plate (*n* = 5 wells for DLX5 siRNA treatment group, *n* = 5 for the siRNA control group) in DMEM++ media and allowed to reach 80% confluency. Cells were transfected with siRNA using Lipofectamine 3000 Reagent (Thermo Fisher Scientific, Waltham, MA, USA) according to the standard manufacturer protocol. Complete growth media or chondrogenesis media was supplemented 24 h post-transfection to analyze basal uninduced and chondrogenesis-induced gene expression after knockdown, respectively. All groups were processed for quantitative mRNA analysis 72 h post-transfection. Protein analysis was carried out at 48 h post-transfection in the complete growth medium.

DLX5 overexpression in C-PCs was performed using DLX5 adenovirus (Cat. No. 085797A, Applied Biological Materials, Richmond, BC, Canada). The transduction procedure was performed according to the manufacturer’s protocol. First, 2.5 × 10^5^ C-PCs were per well were plated into a 6-well plate (*n* = 8 wells for DLX5 adenovirus overexpression group and *n* = 8 wells for control adenovirus group) into DMEM++ media. Once the target cells were allowed to reach ~90% confluency, this was deemed “Day 0”. To begin the transduction, the culture medium was aspirated and 100 µL of the DLX5 adenovirus concentrate was supplemented to cover the cells. Immediately after this, we added 900 µL of DMEM++ media and allowed the cells to incubate for one hour in a 37 °C cell incubator. Next, the media containing the virus was removed and replaced with fresh DMEM++ media. Gene transduction and the downstream effects were then evaluated 48 h after transduction by RT-qPCR. This transduction protocol was also followed for the control group, using a CMV-Null adenovirus (Applied Biological Materials, Richmond, BC, Canada).

### 4.6. Staining of Patient Cartilage

Human patient cartilage from the femoral condyle containing OA lesions (and regions with no lesions) was fixed in 4% formalin, embedded in paraffin, and sectioned into 6 µm thick section. Sections were mounted on glass slides. Antigen retrieval was performed using HistoReveal (Abcam, Cambridge, MA, USA) for 20 min followed by Hyaluronidase (0.01g/mL) treatment for another 20 min at 37 °C. Slides were treated with 0.3% hydrogen peroxide in methanol and blocked for nonspecific staining using Superblock (Thermo Fisher Scientific, Waltham, MA, USA). For immunostaining, slides were stained with the DLX5 rabbit mAb antibody (1:250 dilution, Cat. No.: ab109737; Abcam) overnight at 4 °C. Slides were washed 3 times in 1× tris-buffered saline containing 0.1% Tween 20 (TBST), for 5 min on a shaker. Slides were stained with Horseradish peroxidase (HRP) conjugated goat anti-Rabbit secondary antibody (Bio-Rad, Hercules, CA, USA) for 50 min at room temperature. Slides were washed with 1× TBST, as before, and treated with 3,3′-Diaminobenzidine (DAB) for 2 min. Slides were rinsed under tap water, dehydrated, cleared, and coverslipped. Sister section slides were stained with Safranin-O (Saf-O)/Fast green by 0.04% Fast Green Solution for 5 min followed by a quick rinse with a 1% acetic acid solution. Slides were then stained with 0.1% Saf-O solution for 5 min and mounted using resinous medium. Slides were imaged using the Nikon Eclipse 90i Digital Imaging System.

### 4.7. Apoptotic Cell Detection Using Active Caspase-3/7 Immunostaining

An immunofluorescence caspase3/7 assay was used to examine apoptotic activity in cells. Experiments were performed on a 96-well plate, with an initial plating density of 5 × 10^4^ cells/well. After 48 h in culture, active caspase levels were directly assessed by the CellEvent Caspase-3/7 Green Detection Reagent (Thermo Fisher Scientific, Waltham, MA, USA). 

### 4.8. Western Blotting

Total protein was extracted from DLX5 knockdown in BM-MSCs and nontargeting siRNA control wells in BM-MSCs using 1× RIPA Buffer containing 1mM PMSF (Cell Signaling Technology, Danvers, MA, USA). Cells were washed with sterile cold PBS and later the lysis extract was centrifuged for 10 min at 4 °C. The concentration of total protein supernatant was determined using the Pierce BCA Protein Assay kit Thermo Fisher Scientific, Waltham, MA, USA. A total of 40 ug protein was resolved on 4–15% Mini-PROTEAN^®^ TGX™ Precast Gels (Bio-Rad). Blots were transferred to PVDF membrane (Bio-Rad, Hercules, CA, USA). Blocking of the membrane was done at room temperature for 2 h with 5% Nonfat Dry Milk (Cell Signaling Technology, Danvers, MA, USA) in tris-buffered saline with Tween-20 (0.1%, TBST). Membranes were incubated overnight at 4 °C with specific primary antibodies: 1:200 DLX5 (Cat. No.: ab109737; Abcam), 1:100 Caspase-3 (Cat. No.: 9668S; Cell Signaling Technology) and 1:1000 β-Actin (Cat. No.: 8H10D10; Cell Signaling Technology). Later, membranes were incubated with secondary antibodies: IRDye 680RD Goat anti-Rabbit, IRDye 680RD Goat anti-Mouse, and IRDye 800CW Goat anti-Mouse (LI-COR Biosciences, Lincoln, NE, USA) respectively at 1:5000 for 2 h at room temperature. Membranes were imaged using an Odyssey fluorescence scanner (LI-COR Biosciences, Lincoln, NE, USA).

### 4.9. Statistics

Statistical analysis using a Student’s t-test was performed for experiments when analyzing two groups, or experiments that compared each experimental group to a single control group. One-way analysis of variance (ANOVA) and post hoc analysis was used when comparing more than two individual groups that required comparisons between each group. N ≥ 3 for all experiments. Error bars represent ±1 standard deviation from the mean and *p* ≤ 0.05 was considered statistically significant.

## 5. Conclusions

Our findings indicate that DLX5 expression drives the cellular hypertrophy program in cells of the mesenchymal progenitor cell lineage. It also suggests that DLX5 knockdown attenuates cell hypertrophy without significantly impacting critical early chondrogenesis states of mesenchymal progenitors in order to facilitate the therapeutic reprogramming of BM-MSCs. We believe that these findings will contribute towards developing novel and effective strategies to optimally prime cells for cartilaginous tissue repair. 

## Figures and Tables

**Table 1:** List of forward and reverse primers, in 5′ to 3′ orientation, used for RT-qPCR.

Gene	Forward Seq.	Reverse Seq.	Accession
*ALPL*	CTGGACGGACCCTCGCCAGTG	TGCAATCGACGTGGGTGGGAGG	NM_000478.5
*Beta-Actin*	GGACCTGACTGACTACCTCAT	CGTAGCACAGCTTCTCCTTAAT	NM_001101.4
*BGLAP*	AGCAAAGGTGCAGCCTTTGT	GGCTCCCAGCCATTGATACA	NM_199173.6
*COL1A1*	CAGGAGGCACGCGGAGTGTG	GGCAGGGCTCGGGTTTCCAC	NM_000088.3
*COL2A1*	CTCCCAGAACATCACCTACCACT	CGTGAACCTGCTATTGCCCT	NM_001844.4
*COL10A1*	GCCCACAGGCATAAAAGGCCC	GAAGGACCTGGGTGCCCTCGA	NM_000493.3
*DLX5*	ACAGCCATGTCTGCTTAGACC	AGACGGATGGTGCATAGCTG	NM_005221.6
*MMP13*	ATGCGGGGTTCCTGATGTGG	GGCCCAGGAGGAAAAGCATG	NM_002427.4
*RUNX2*	CTCTGACTTCTGCCTCTGGC	GGTGTGGTAGTGAGTGGTGG	NM_001024630.4

**Table 2:** Cell surface markers utilized for immunophenotyping.

Cell Surface Marker	Description of Marker
CD166	Mesenchymal stem cell/progenitor marker [45]
CD73	Multipotent mesenchymal stromal cell marker [19]
CD90	Multipotent mesenchymal stromal cell marker [19]
CD105	Multipotent mesenchymal stromal cell marker [19]
CD106/VCAM1	Vascular cell adhesion marker [45]
CD45	Hematopoietic cell marker [45]
CD34	Hematopoietic stem/progenitor cell and endothelial marker [46]
CD14	Monocyte/macrophage and hematopoietic marker [47,48]
CD11b	Macrophage/monocyte cell marker [49]

## Supplementary Materials

The following are available online at https://www.mdpi.com/1422-0067/21/14/4823/s1.
