# Supplementary material for: Distal-Less Homeobox 5 Is a Therapeutic Target for Attenuating Hypertrophy and Apoptosis of Mesenchymal Progenitor Cells
Source: Int J Mol Sci. 2020 Jul 8;21(14):4823. doi: 10.3390/ijms21144823 (PMC7404054; doi:10.3390/ijms21144823)
Supplement: Supplementary file 1 [file ijms-21-04823-s001.pdf]

GENE	baseMean	log2FoldChange	lfcSE	stat	pvalue	padj
DLX5	54.76529868	7.665545782	1.042593546	7.352381768	1.95E-13	5.61E-13
COL10A1	273.894427	7.178912848	0.401507646	17.87989077	1.69E-71	1.90E-70
COL1A1	735856.4417	6.308282821	0.060270059	104.6669424	0	0
MMP11	63.83895987	5.537707415	0.510068285	10.85679619	1.85E-27	8.68E-27
MMP13	22.99523852	5.390433956	0.801362134	6.726589304	1.74E-11	4.54E-11
BMP2	509.5580181	3.92979212	0.142200819	27.63550973	4.17E-168	1.28E-166
RUNX2	636.9932357	3.711846276	0.114000412	32.5599374	1.51E-232	7.48E-231
BMP4	335.4568653	3.260371348	0.13934004	23.39866803	4.41E-121	9.05E-120
ALPL	14.9799022	2.584918569	0.545502604	4.73859987	2.15E-06	4.15E-06
IL6	278.9362908	0.664442652	0.13308584	4.992587138	5.96E-07	1.19E-06

Supplemental Table 1. Raw base transcript readings, fold-changes, and p-values in genes of interest that are upregulated in BM-MSCs Vs. C-PCs. Data was obtained using RNA-Seq.
